# Supplementary material for: Endosomally Localized RGLG-Type E3 RING-Finger Ligases Modulate Sorting of Ubiquitylation-Mimic PIN2
Source: Int J Mol Sci. 2022 Jun 17;23(12):6767. doi: 10.3390/ijms23126767 (PMC9224344; doi:10.3390/ijms23126767)
Supplement: Supplementary file 1 [file ijms-23-06767-s001.zip › ijms-1571276-supplementary.pdf]

Supplementary Information:

Endosomally localized RGLG-type E3 RING-finger ligases modulate sorting of ubiquitylation-mimic PIN2

Katarzyna Retzer [1, §], Jeanette Moulinier-Anzola [1], Rebecca Lugsteiner[1], Nataliia Konstantinova[1,#], Maximilian Schwihla [1], Barbara Korbei[1,*] and Christian Luschnig[1,*]

1 University of Natural Resources and Life Sciences, Vienna, Department of Applied Genetics and Cell Biology, Institute of Molecular Plant Biology, Muthgasse 18, 1190 Vienna, Austria

§ present address: Institute of Experimental Botany of the Czech Academy of Sciences, Rozvojová 263 Praha 6, Czech Republic

# present address: VIB-UGent Center for Plant Systems Biology, Technologiepark 71, 9052 Gent, Belgium

* Correspondence: barbara.korbei@boku.ac.at (B.K.); christian.luschnig@boku.ac.at (C.L.)

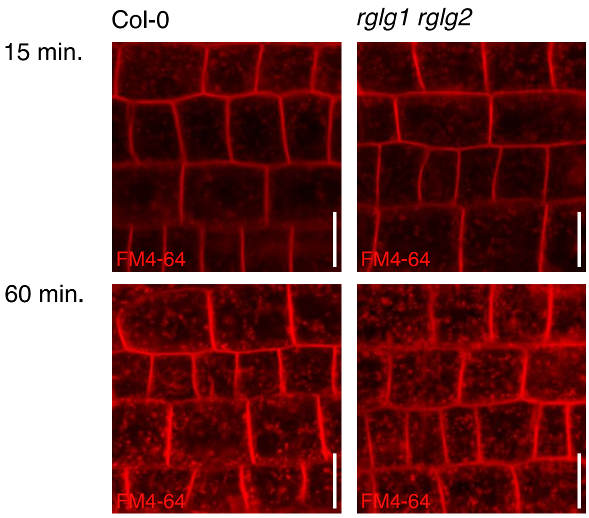

Figure S1: Kinetics of endocytic sorting monitored, by following uptake and internalization of FM4-64 fluorescent dye (red signals) in *rglg1 rglg2* (right panels) and wild-type (Col0; left panels) at 15 min. and 60 min. Representative images are shown. Scale bars: 10 μm.

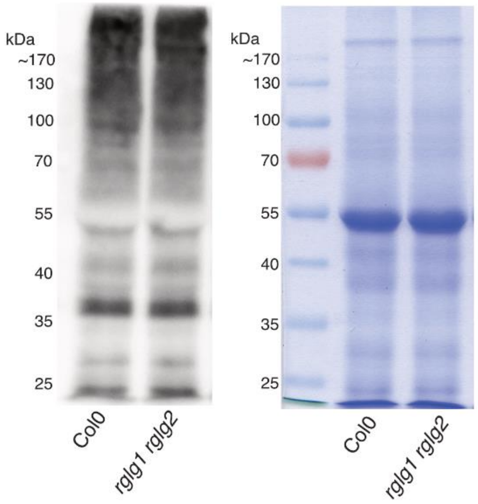

Figure S2: Accumulation of ubiquitin conjugates in Col0 and *rglg1 rglg2*. Total protein extracts of 6-day-old Col0 or *rglg1 rglg2* seedlings lines were subjected to SDS–PAGE followed by immunoblotting using an anti-ubq antibody (P4D1; Santa Cruz Biotechnology; left panel). Coomassie staining for visualization of protein levels in samples used, is shown in the right panel. Migration of a protein size marker is shown in kDa (left).

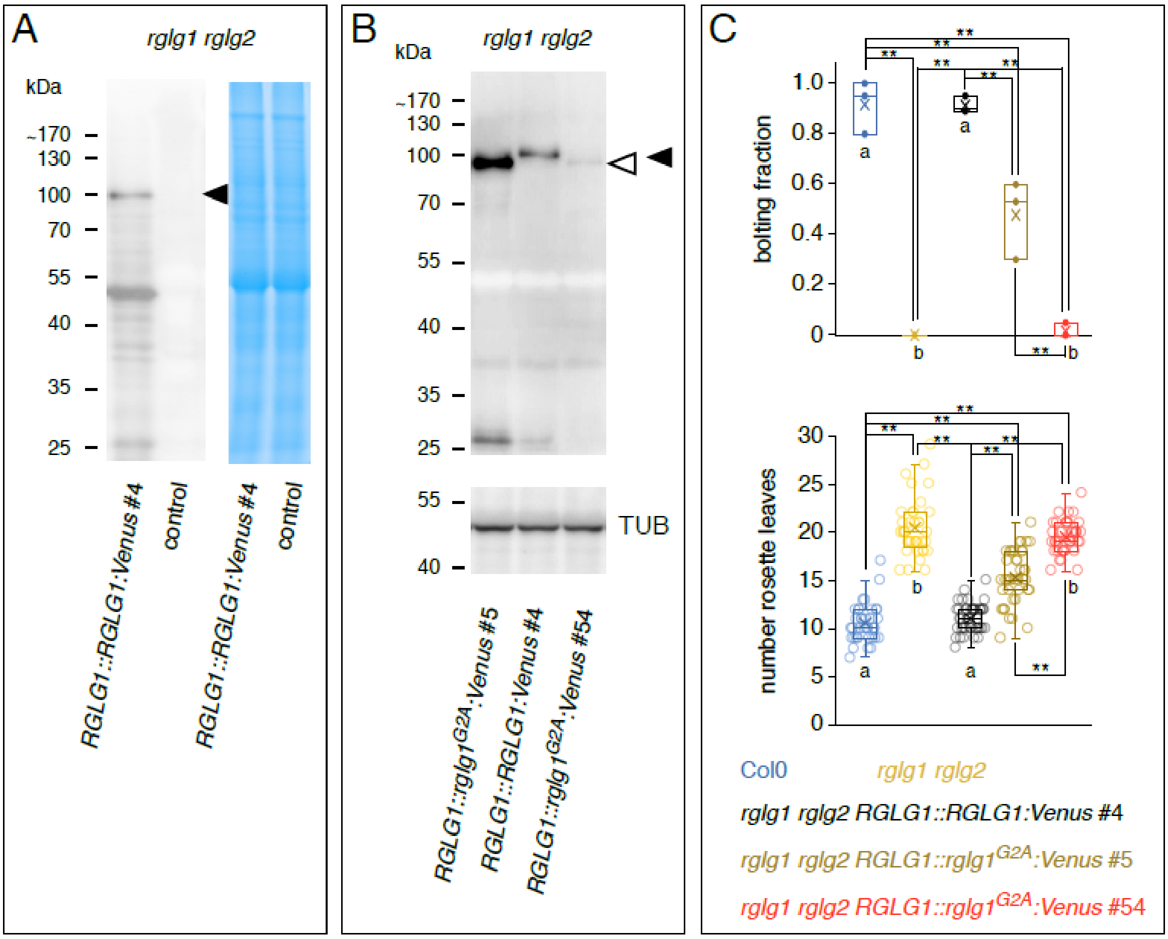

Figure S3: Expression and phenotypes of RGLG1-reporter lines. (A) Expression of RGLG1::RGLG1:Venus #4 in total protein extracts from rglg1 rglg2 at 6 days after germination (black arrowhead). Total root protein extracts from rglg1 rglg2 served as control. Coomassie staining of samples loaded is shown to the right. (B) Comparison of reporter protein levels in total protein extracts from 6-day-old RGLG1:Venus and rglg1G2A:Venus reporter lines probed with anti-GFP (top). Black arrowhead: RGLG1:Venus; open arrowhead: rglg1G2A:Venus; α-tubulin (TUB) served as loading control (bottom). (C) Phenotypic traits of wild type, rglg1 rglg2, rglg1 rglg2 RGLG1::RGLG1:Venus and rglg1 rglg2 RGLG1::rglg1G2A:Venus lines at the stage of flowering. Top graph: Fraction of bolting plants at 24 days after germination. In total 58-to-60 plants were analyzed for each genotype in three biological repeats. Bottom graph: Number of rosette leaves at 32 days after germination. In total 36-to-39 plants were analyzed in three biological repeats. Circles represent single data points; boxes: first and third quartiles; center line: median; 'x': mean value. One-way ANOVA with post-hoc Tukey HSD was performed; ** $p < 0.01$; a,b: $p > 0.05$.

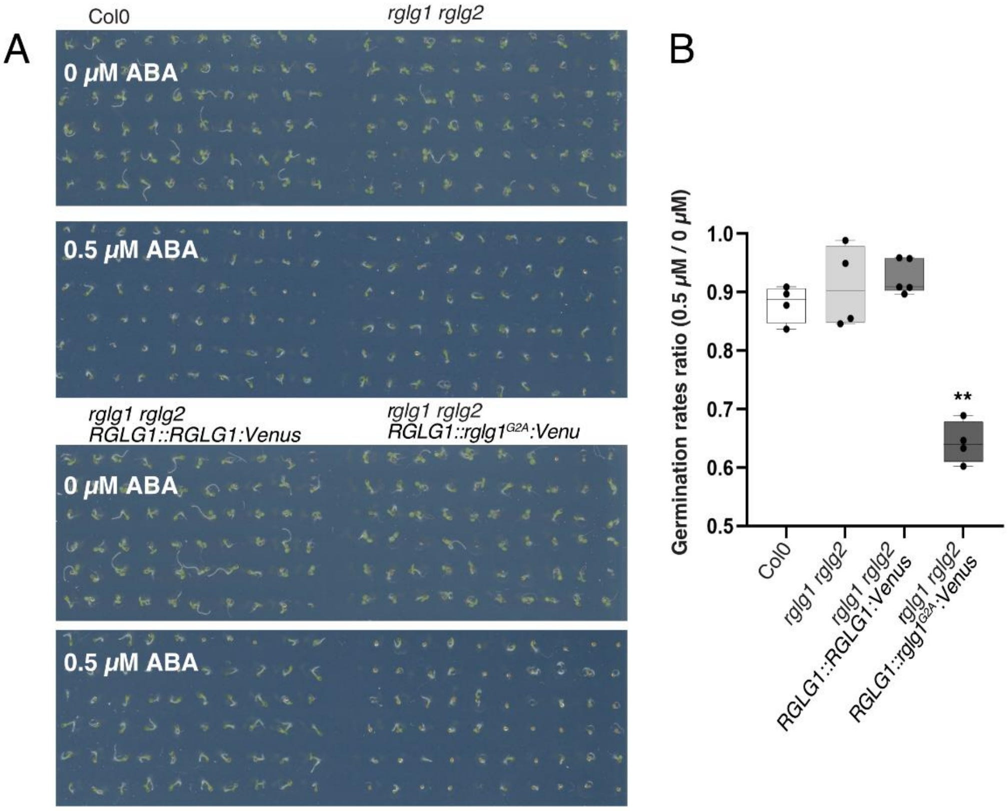

Figure S4: ABA germination assay. **(A)** Seed germination of Col0, *rglg1 rglg2*, *rglg1 rglg2 RGLG1::RGLG1:Venus* and *RGLG::rglg1*^{G2A}*:Venus* after 72 hours on ½ MS plates supplemented with no (0 µM) or 0.5 µM ABA. Expression of *rglg1*^{G2A}*:Venus* in *rglg1 rglg2* (bottom right) leads to enhanced sensitivity to ABA-mediated inhibition of germination compared with *rglg1 rglg2 RGLG1::RGLG1:Venus* (bottom left panel), wild type (upper left panel) or *rglg1 rglg2* (top right panel). **(B)** Germination rate ratios were scored as the percentage of seeds that showed radicle emergence at 48h on ½ MS plates supplemented with 0.5 µM ABA with respect to 0 µM ABA. (100 seeds for each genotype and condition were counted for each experiment; experiments were repeated 4-5 times) Circles represent single data points; boxes: first and third quartiles; center line. Two-tailed t-test was employed to determine statistical significance; ** $P < 0.01$.

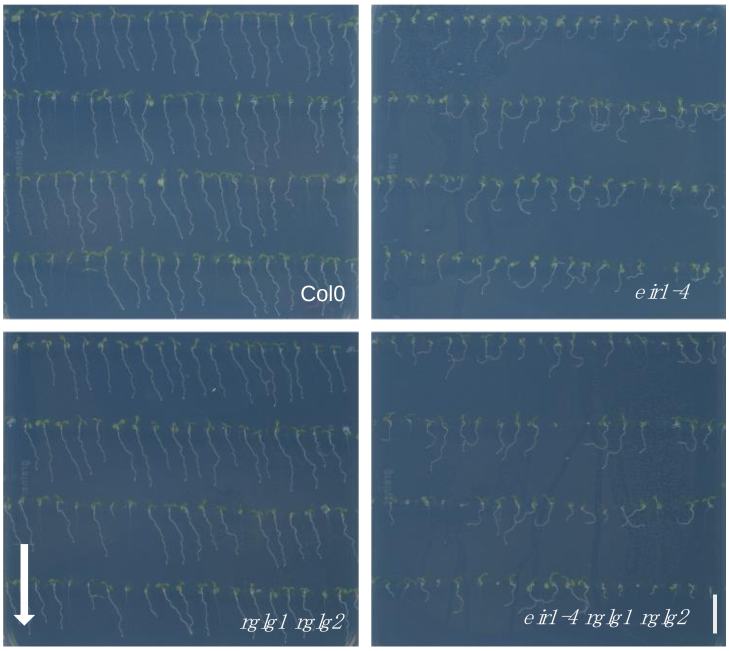

Figure S5: Comparison of directional root growth in Col0, *eir1-4, rglg1 rglg2,* and *eir1-4 rglg1 rglg2* seedlings 5 days after germination. White arrow indicates the direction of the gravity vector. Size bar: 1cm.

A

```
RGLG4 ------------------------------------------------------------------------------------------ 0
RGLG3 ------------------------------------------------------------------------------------------ 0
RGLG5 MLEVLQAIVLFILFWVWLSWMGGSSSKESPRGGGS------------------------------------------------------- 35
RGLG1 --------------------MGGNSKE-------------ESSSPSSSSWASHQSYPQYGPDSYNYPPPPTYAPAPSPAPAPAPVPAPSP 58
RGLG2 --------------------MGTGNSKENWRQSSFRSTSASSASPSSSSWASQQSYPQYGAESYNYPPPPSYAQPPEYTQ---------- 56

RGLG4 -MTMG-----------------NFLKRFGSGKSRSSRNM-TLGTTSSQSHEPSPSDPSL-SLADNTNATKKKYALIPDRFSSLDQVSKAL 70
RGLG3 --------------------------------------------------------------MNMSNKRNQQPSYIADHFSSLDQVITSL 28
RGLG5 ---------------------GRRYERSVSGSSSYSSAWDQSSYYQTPNHPSASPVSSYNSGRQTPKNLERKYSRIADNYRSIDEVTAAL 104
RGLG1 ASSYGPQYSQEGYASQPNNPPPPTYAPAPSPASSYGHQYSQEGYASAASQPNYPPPPSQS-QVADRKKFDRRYSKISDNYSSLLQVSEAL 147
RGLG2 -------------------PPPPLYSTQPYS------------------APSYSAPPSQSYGSDNKKRLERKYSKISDDYSSLEQVTEAL 113
```

B

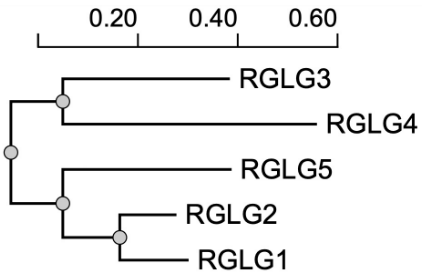

Figure S6: Phylogenetic relationship among the 5 members of the *Arabidopsis* RGLG protein family. **(A)** Multiple sequence alignments of the predicted N-terminal portions of Arabidopsis RGLGs. Glycines that appear myristolyated in RGLG1 and RGLG2 are indicated in red. This peptide domain seems conserved in RGLG5, although not found at the very N-terminus of the predicted RGLG5 ORF. The myristoylation motif cannot be detected in the predicted RGLG3 and RGLG4 ORFs. Alignments were performed with ClustalX by using default settings. **(B)** Alignment of predicted full-length RGLG ORFs was used for determination of phylogenetic distances, by employing the FastME 2.1.6 tool (https://bioweb.pasteur.fr/packages/pack@fastme@2.1.6.1; Lefort et al., 2015, *Molecular Biology and Evolution*; doi.org/10.1093/molbev/msv150). Branch length is indicated on top. Statistical support for branches was calculated with bootstrap replicas (n = 100).
